# Supplementary material for: Targeting RAD52 overcomes PARP inhibitor resistance in preclinical Brca2-deficient ovarian cancer model
Source: bioRxiv. 2025 Sep 26:2025.09.24.678351. Preprint. [Version 1] doi: 10.1101/2025.09.24.678351 (PMC12485677; doi:10.1101/2025.09.24.678351)
Supplement: Supplement 1 — Supplementary Figure 1. Flow diagram of patient selection for the tissue microarray (TMA). A total of 137 patients with high-grade serous ovarian cancer were initially considered. From this cohort, 26 patients were excluded due to insufficient tumor volume (tumor area <5% of each core), 20 patients whose samples were from recurrent tumors were excluded, and 19 patients were excluded because they had received neoadjuvant therapy, which could potentially alter the molecular profile. This resulted in a final cohort of 72 patients for the analysis. Supplementary Figure 2. Evaluation of Brca2 deletion using next-generation sequencing (a) DNA sequencing of mouse Brca2 exon 3 in ID8-P, ID8-PB, and ID8-OR cell lines. (b) DNA sequencing of mouse Brca2 exon 11 in HGS2 and HGS2-OR cell lines. Supplementary Figure 3. Generation and validation of Rad52 knockout ID8-OR cells. (a) Schematic illustrating the generation of Rad52-depleted PARP inhibitor (PARPi) resistant ovarian cancer cells. (b) Schematic of the RAD52 domain structure, showing the DNA-, RPA-, and RAD51-binding domains, and a nuclear localization signal (NLS). The CRISPR/Cas9-mediated truncation occurs at amino acid 68. (c) Guide RNA target sequence in exon 4 of the mouse Rad52 gene and the corresponding CRISPR/Cas9 cleavage site. (d) Amplicon sequencing of the target region in wild-type (Scramble) and Rad52 knockout monoclonal cells. (e) Quantitative validation of RAD52 knockout by Western blot. Data are presented as mean ± standard deviation from three independent experiments, with individual data points shown for each biological replicate. Statistical significance was assessed by two-sided Student’s t-tests for two-group comparisons and one-way ANOVA followed by Šídák’s multiple comparisons test for comparisons among three groups. KO, knockout; RNP, ribonucleoprotein-based; KD, knockdown; Scr, scrambled; shRNA, short hairpin RNA. Supplementary Figure 4. Tumor burden and ascites volume at different time points in m [file media-1.pdf]

Supplementary Figure 1

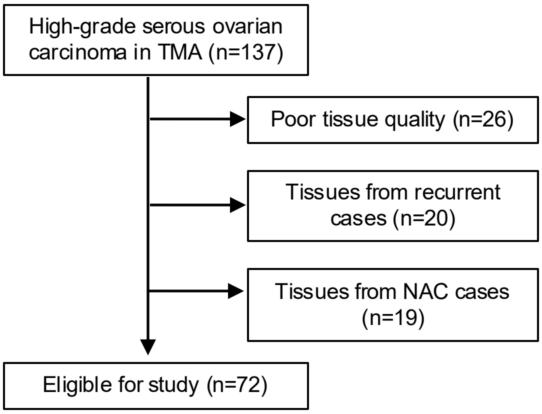

Supplementary Figure 2

Site of the *Brca2* mutation

a

ID8-P
Brca2 wt,
Refference

NM_001081001.2:c.=, NP_001074470.1:p.(=)

exon2 | exon3

agatgcagcacagcagatttaggaccgataagcctcaattggtttgaggagctttcctca
R C S T A D L G P I S L N W F E E L S S
gaagccccccatacaattctgaacctccggaggaatctgagtataagccccacggttat
E A P P Y N S E P P E E S E Y K P H G Y
gaaccacagctgtttaaaacaccacagaggaatccccctaccatcagtttgcttcaact
E P Q L F K T P Q R N P P Y H Q F A S T
ccaataatgttcaaa
P I M F K

ID8-PB/OR
Brca2 del

NM_001081001.2:c.[69T>A;94_219del] NP_001074470.1:p.[Asp23Glu;Phe32_Gln73del]

Allele 1

exon2 | exon3

agatgcagcacagcagaattaggaccgataagcctcaattgg-------------------
R C S T A E L G P I S L N W

---tttgcttcaact
F A S T
ccaataatgttcaaa
P I M F K

NM_001081001.2:c.169del NP_001074470.1:p.Tyr57Metfs*23

Allele 2

exon2 | exon3

agatgcagcacagcagatttaggaccgataagcctcaattggtttgaggagctttcctca
R C S T A D L G P I S L N W F E E L S S
gaagccccccatacaattctgaacctccggaggaatctgagtataagccccacggt-at
E A P P Y N S E P P E E S E Y K P H G M
gaaccacagctgtttaaaacaccacagaggaatccccctaccatcagtttgcttcaact
N H S C L K H H R G I P P T I S L L Q L
ccaataatgttcaaa
Q *

b

Brca2 wt
reference

NM_001081001.2:c.=, NP_001074470.1:p.=

exon10 | exon11 | exon12

aatgtaaattcaggtataccagattcttct.........gcagttggacaacccccaatcaaaaga
N V N S G I P D S S A V G Q P P I K R

HGS, HGS2-OR
Brca2del

NM_001081001.2:c.1877_6685del, NP_001074470.1:p.Gly626_Val2228del

exon10 | exon12

aatgtaaattcaggacaacccccaatcaaaaga
N V N S G Q P P I K R

Supplementary Figure 3

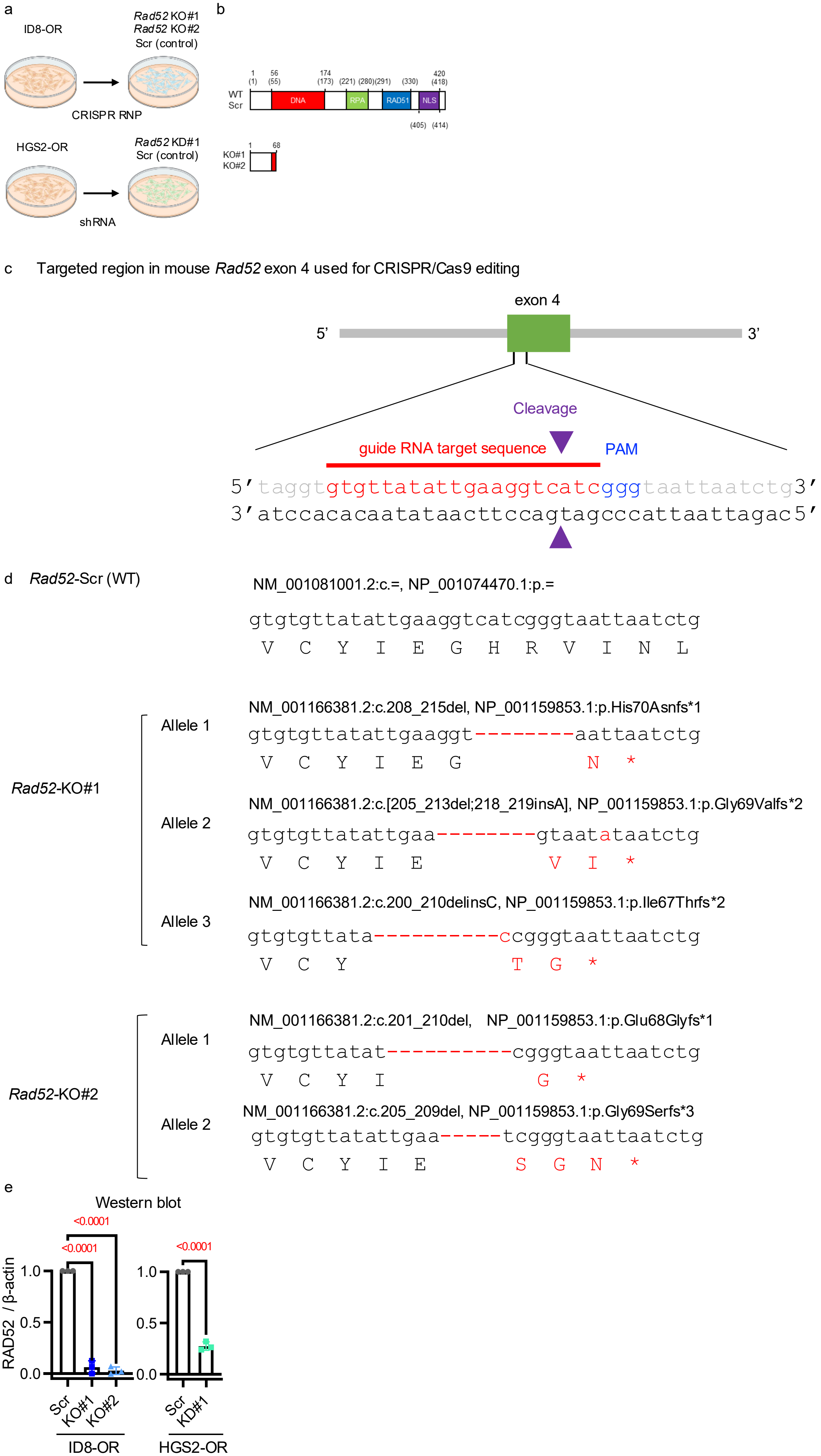

Supplementary Figure 4

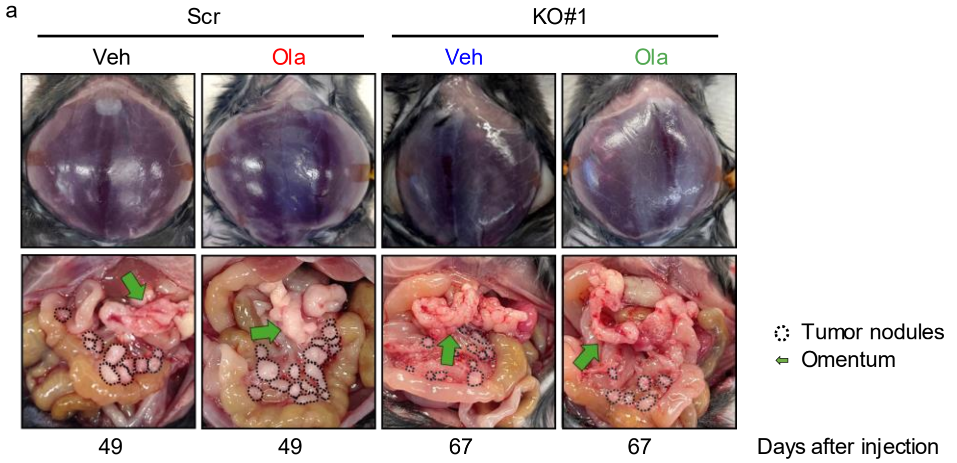

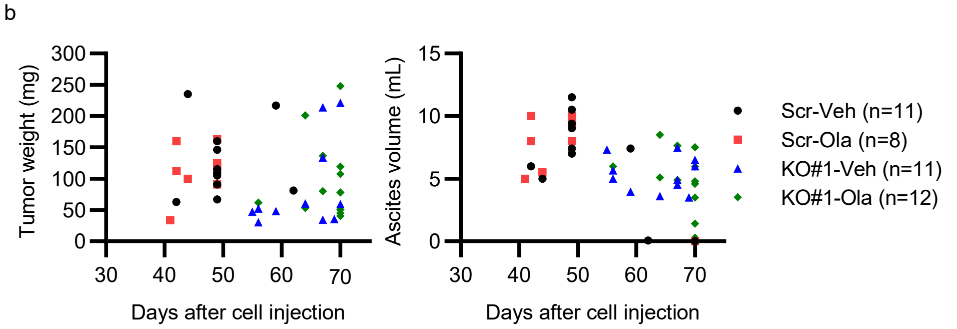

Supplementary Figure 5

a

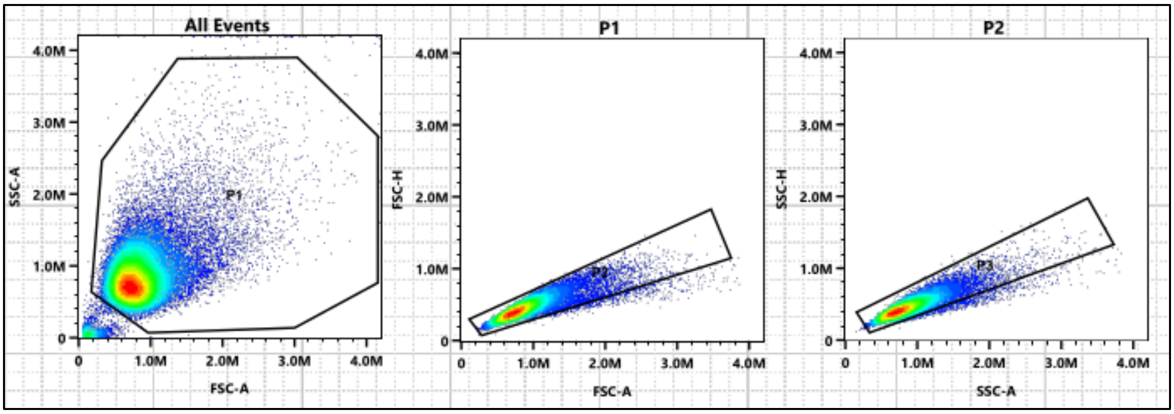

b SSA reporter assay. hprtSAGFP(+), pCBASceI (+). ID8-OR, ID8-PB, ID8-P

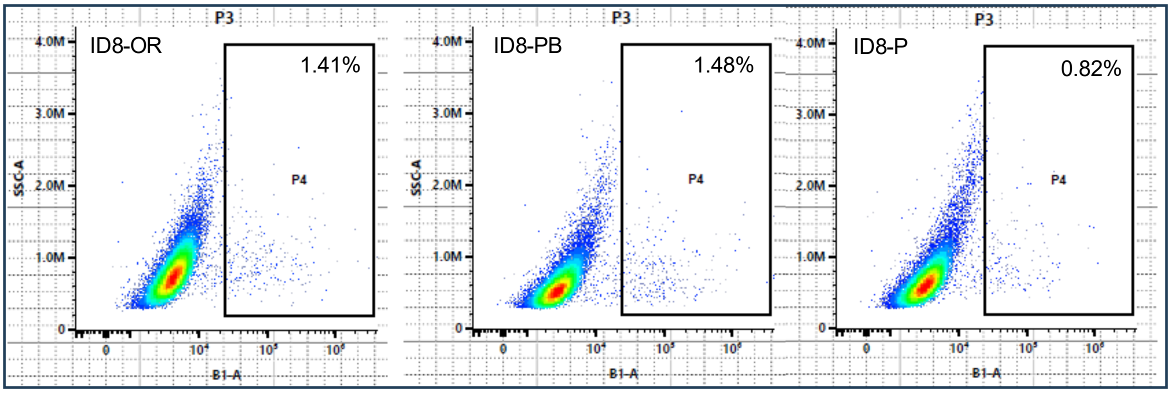

hprtSAGFP(+), pCBASceI (-). ID8-OR, ID8-PB, ID8-P

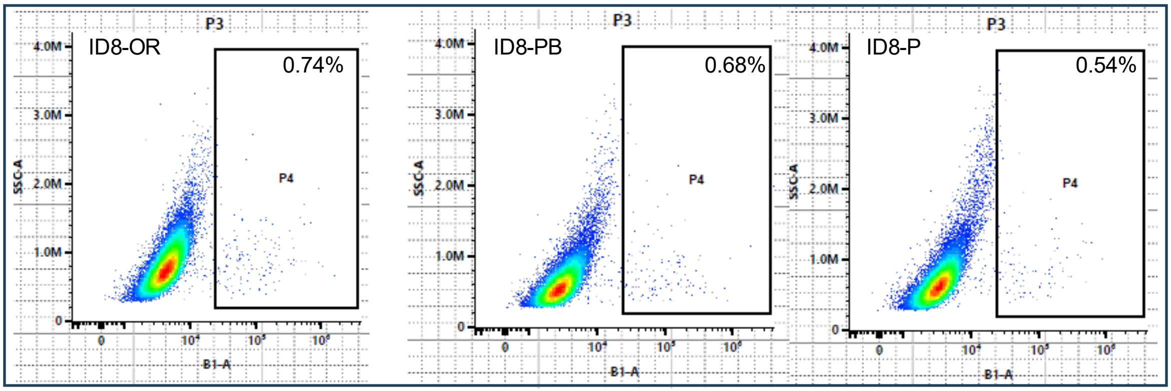

c SSA reporter assay. hprtSAGFP(+), pCBASceI (+). HGS2-OR, HGS2

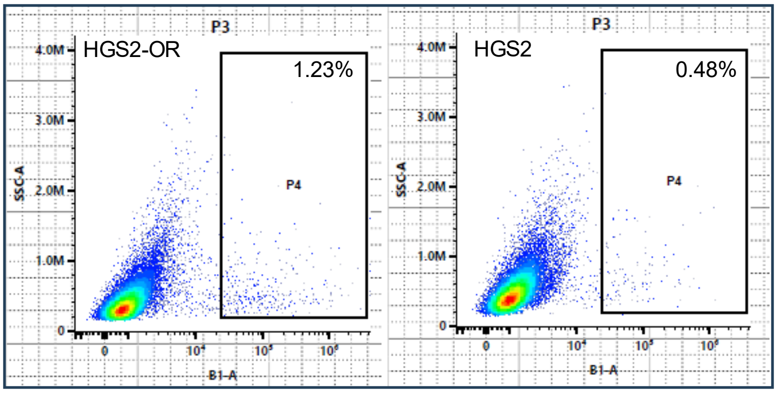

hprtSAGFP(+), pCBASceI (-). HGS2-OR, HGS2

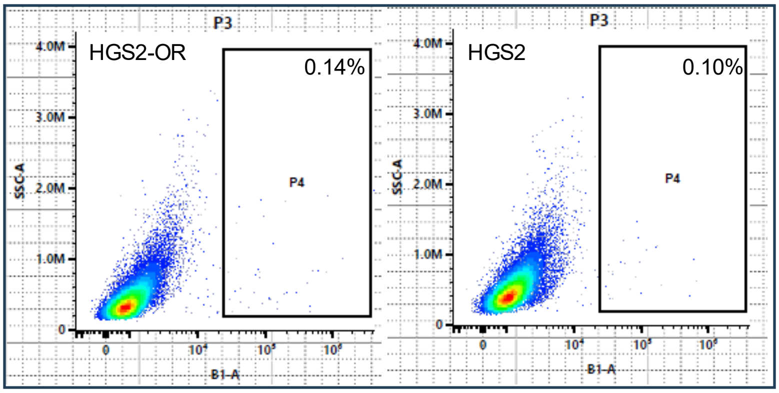

d SSA reporter assay. hprtSAGFP(+), pCBASceI (+). ID8-Scr, ID8-KO#1, ID8-KO#2

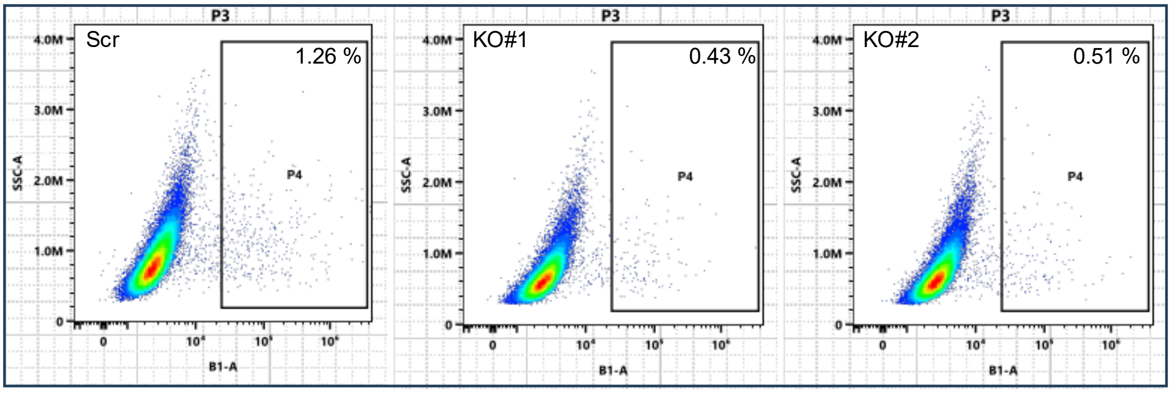

hprtSAGFP(+), pCBASceI (-). ID8-Scr, ID8-KO#1, ID8-KO#2

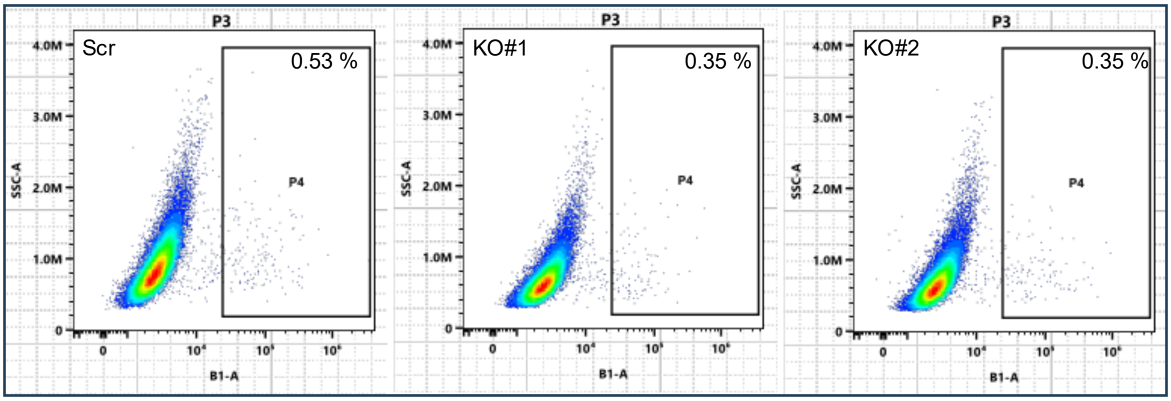

e SSA reporter assay. hprtSAGFP(+), pCBASceI (+). HGS2-Scr, HGS2-KD#1

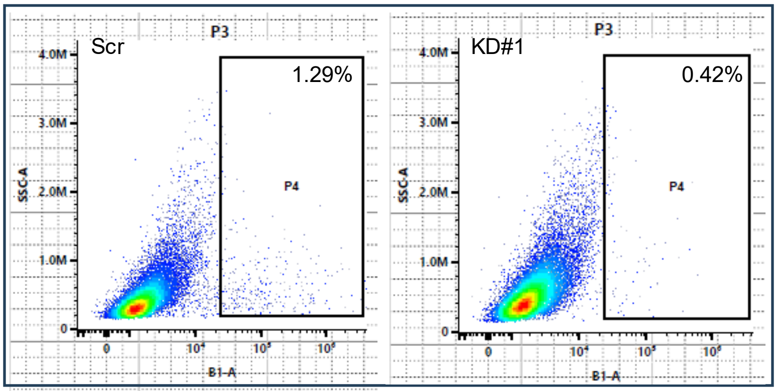

hprtSAGFP(+), pCBASceI (-). HGS2-Scr, HGS2-KD#1

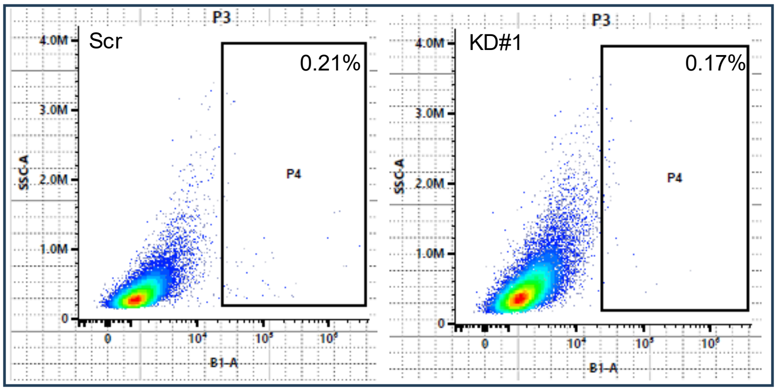

f HR reporter assay. pDR-GFP(+), pCBASceI (+). ID8-OR, ID8-PB, ID8-P

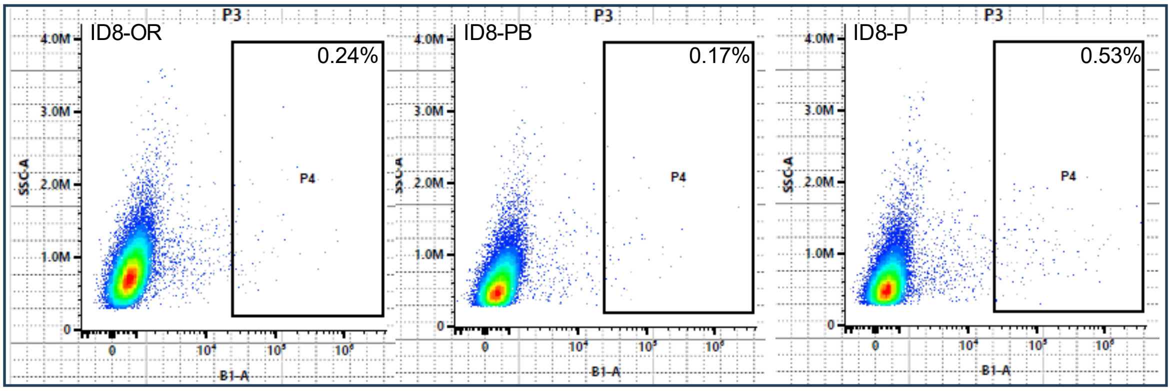

pDR-GFP(+), pCBASceI (-). ID8-OR, ID8-PB, ID8-P

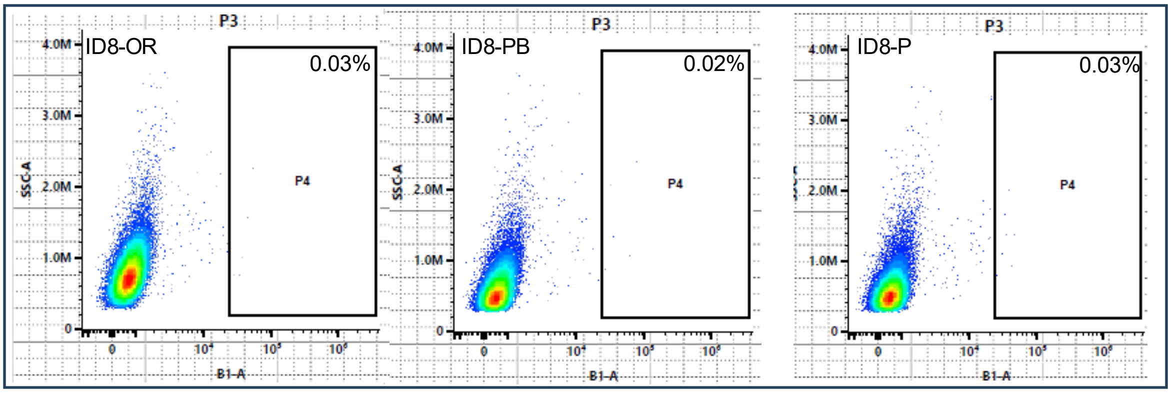

g HR reporter assay. pDR-GFP(+), pCBASceI (+). HGS2-OR, HGS2

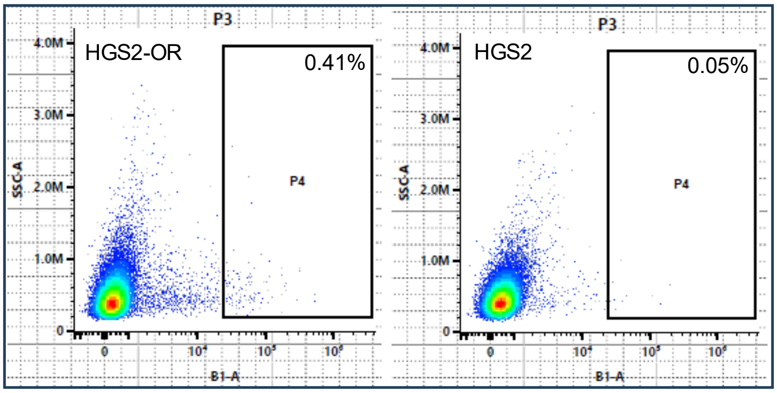

pDR-GFP(+), pCBASceI (+). HGS2-OR, HGS2

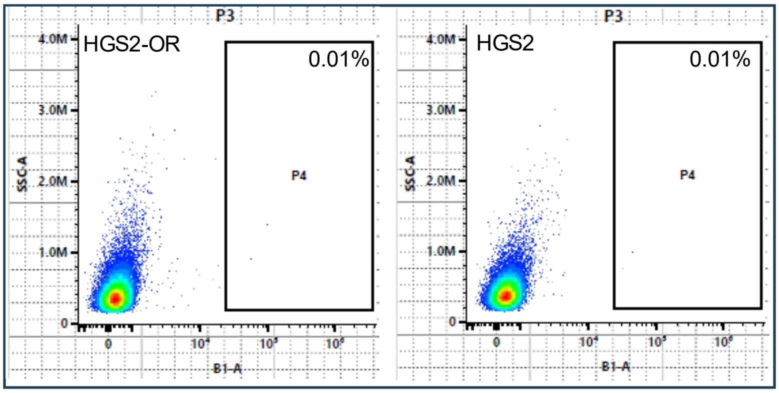

h HR reporter assay. pDR-GFP(+), pCBASceI (+). ID8-Scr, ID8-KO#1, ID8-KO#2

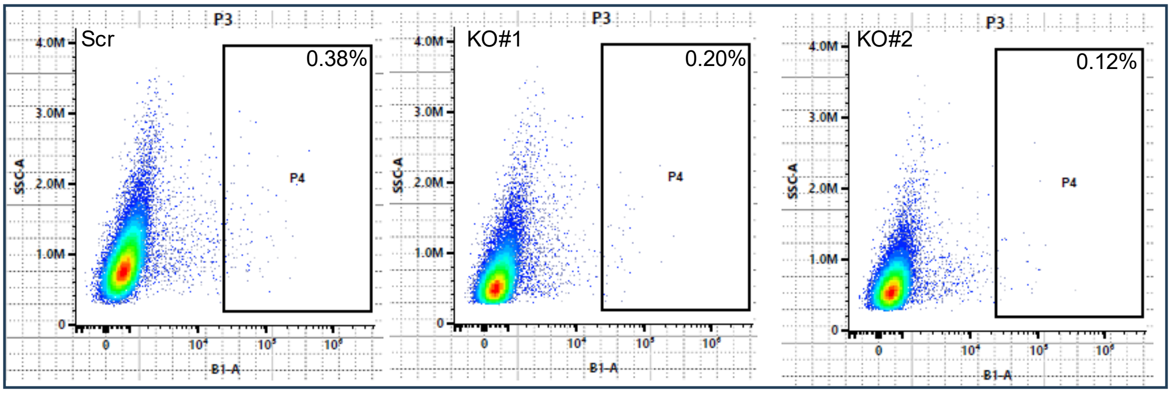

pDR-GFP(+), pCBASceI (-). ID8-Scr, ID8-KO#1, ID8-KO#2

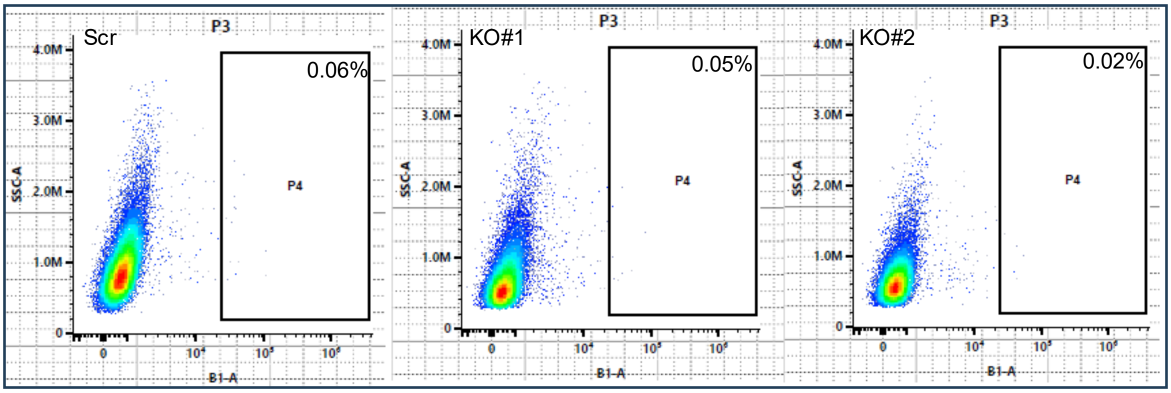

i HR reporter assay. pDR-GFP(+), pCBASceI (+). HGS2-Scr, HGS2-KD#1

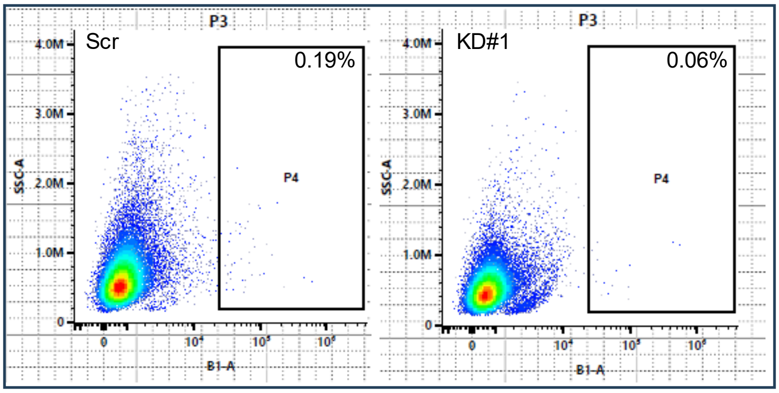

pDR-GFP(+), pCBASceI (-). HGS2-Scr, HGS2-KD#1

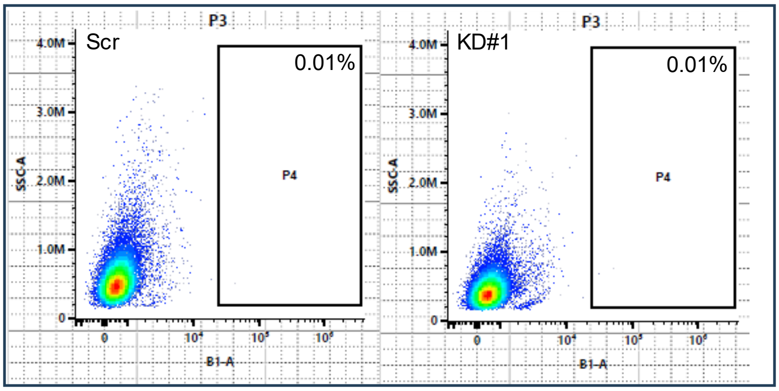

j Untransfected. ID8-P, ID8-PB, ID8-OR

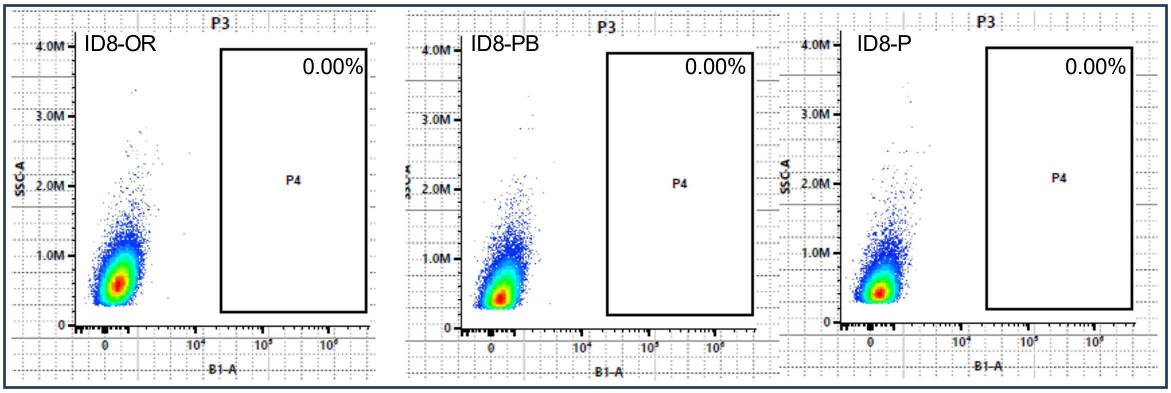

Untransfected. HGS2, HGS2-OR

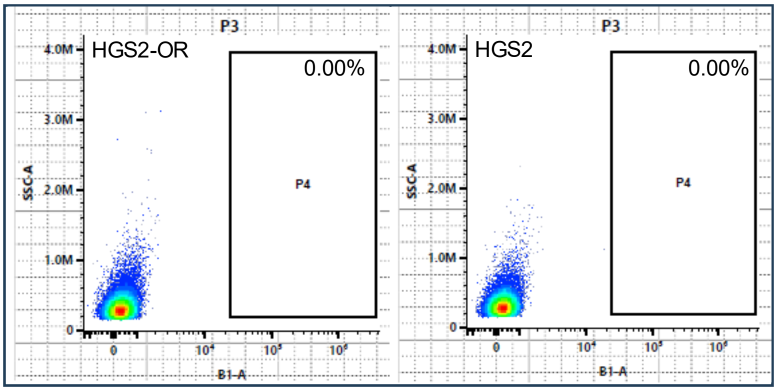

Untransfected. ID8-Scr, ID8-KO#1, ID8-KO#2

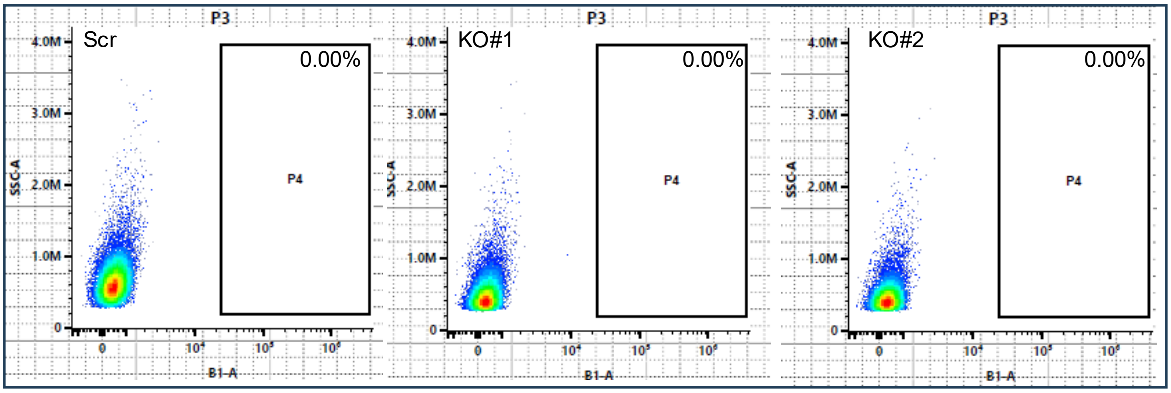

Untransfected. HGS2-Scr, HGS2-KD#1

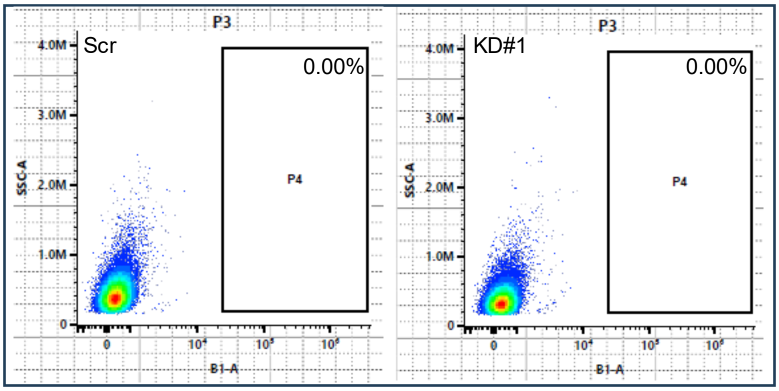

Supplementary Figure 6

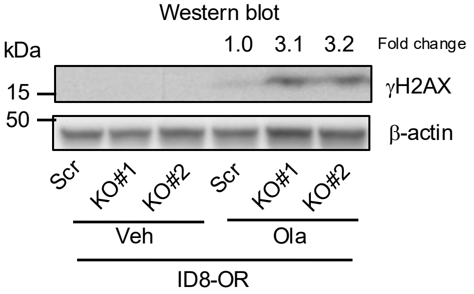

Supplementary Figure 7

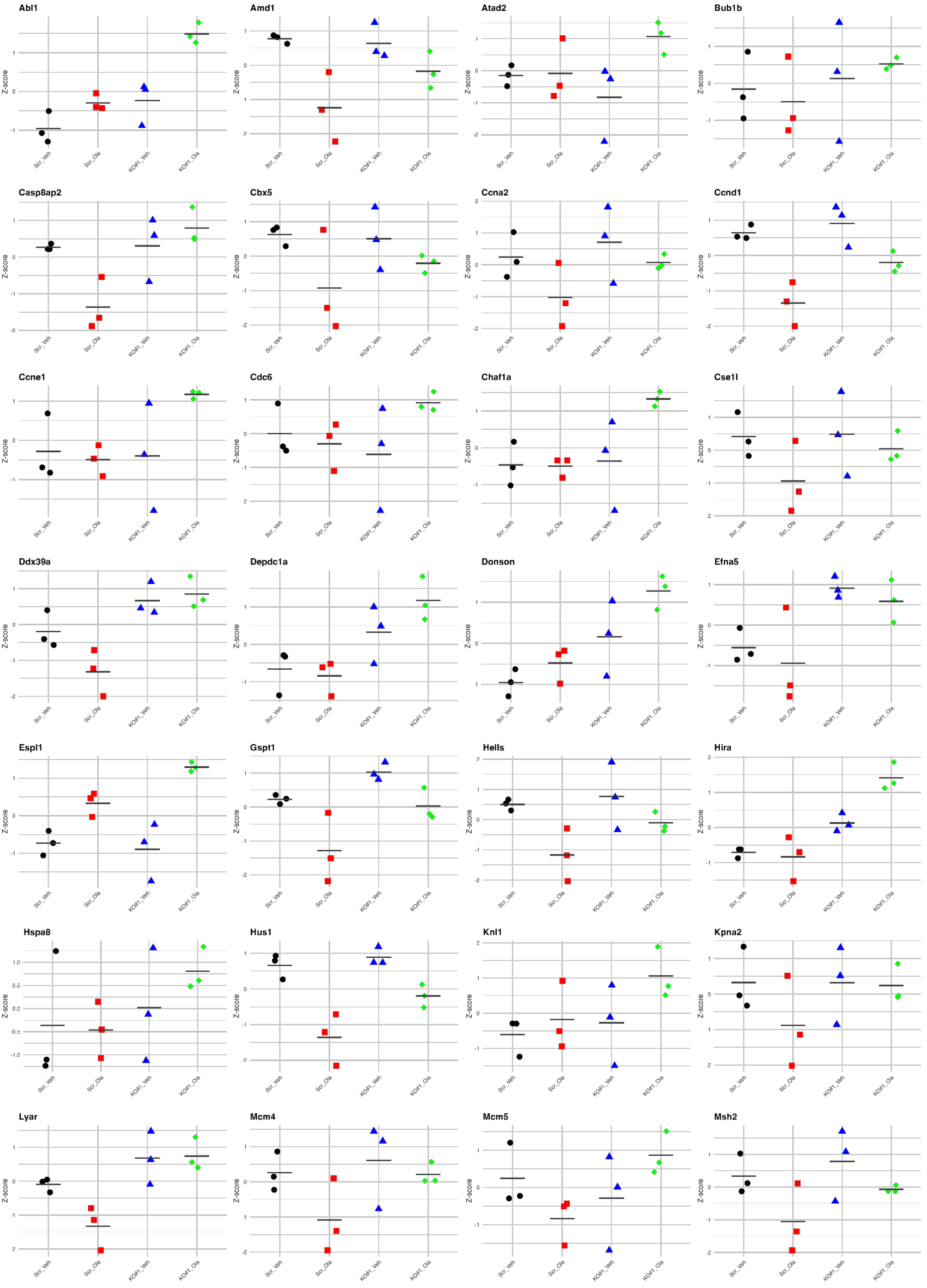

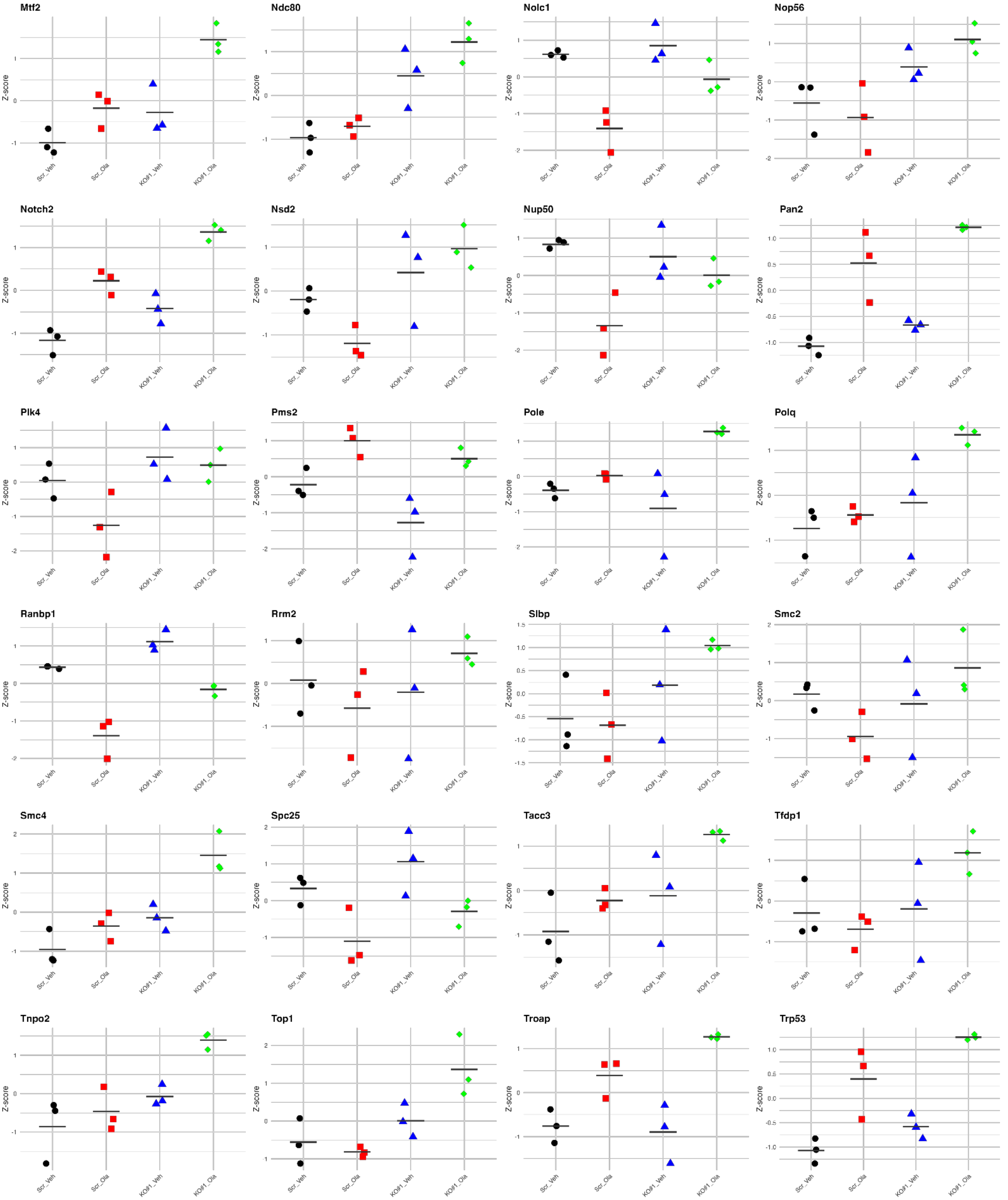

Mtf2
Ndc80
Nolc1
Nop56
Notch2
Nsd2
Nup50
Pan2
Plk4
Pms2
Pole
Polq
Ranbp1
Rrm2
Slbp
Smc2
Smc4
Spc25
Tacc3
Tfdp1
Tnpo2
Top1
Troap
Trp53
Z-score
Scr_Veh
Scr_Ola
KO#1_Veh
KO#1_Ola

Supplementary Figure 8

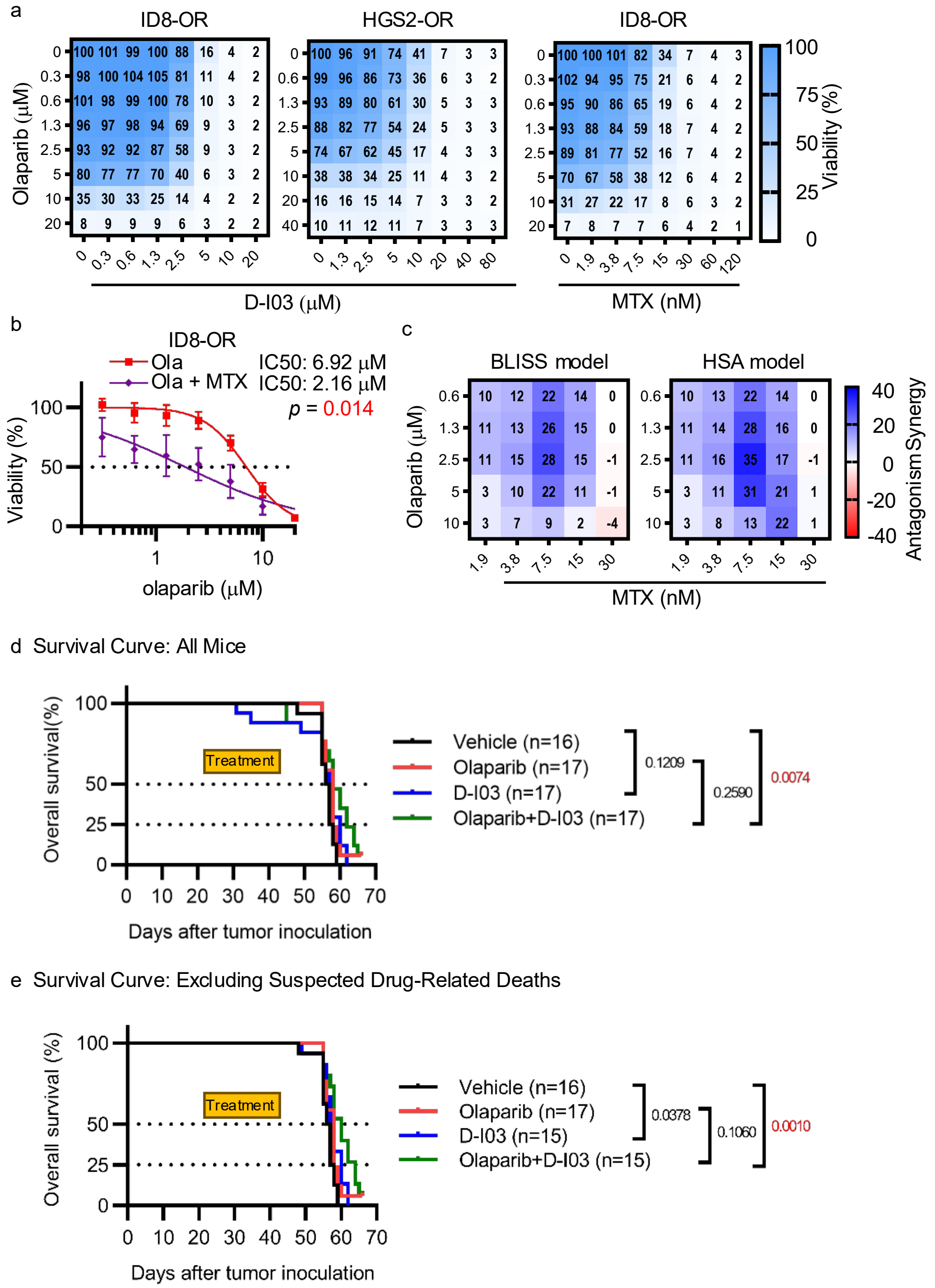

Supplementary Table 1: Patient characteristics of TMA cohort.

	Total (n=72)	RAD52 low (n=36)	RAD52 high (n=36)	P-value
Age (years)	59.6 ± 10.2	59.9 ± 9.8	59.4 ± 10.8	0.83
FIGO stage				0.20
I-II	3 (4.2)	3 (8.3)	0 (0)	
III-IV	64 (88.9)	30 (83.3)	34 (94.4)	
Missing	5 (6.9)	3 (8.3)	2 (5.6)	
BRCA Mutation				0.30
None	32 (44.4)	13 (36.1)	19 (52.8)	
BRCA	19 (26.4)	12 (33.3)	7 (19.4)	
BRCA1	10	6	4	
BRCA2	2	2	0	
BRCA1+2	1	0	1	
No data	6	4	2	
No testing/Missing	21 (29.2)	11 (30.6)	10 (27.8)	
Neoadjuvant Chemotherapy				
No	72 (100)	36 (100)	36 (100)	
Cytoreduction Status				>0.99
Optimal	55 (76.4)	28 (77.8)	27 (75.0)	
Suboptimal	13 (18.1)	6 (16.7)	7 (19.4)	
Missing	4 (5.6)	2 (5.6)	2 (5.6)	
PARP inhibitor Use				0.31
YES	22 (30.6)	13 (36.1)	9 (25.0)	
NO	49 (68.1)	22 (61.1)	27 (75.0)	
Missing	1 (1.4)	1 (2.8)	0 (0)	
Median Follow-up Months (IQR)	64.0 (37.8–89.8)	68.0 (50.3–89.8)	59.0 (25.3.3–90.5)	0.22

Data are n (%) unless stated otherwise. ± Denotes standard deviation Statistical analyses were performed as follows: age was compared using the Student's t-test; follow-up duration was analyzed using the Mann–Whitney U test; and categorical variables were compared using Fisher's exact test. IQR, Interquartile Range.

Supplementary Table 2 Cell lines used in this study.

Cell line	Lineage	Genetic background	Notes	Ref.
ID8-P	Mouse	*Trp53*$^{-/-}$		82
ID8-PB	Mouse	*Trp53*$^{-/-}$, *Brca2*$^{-/-}$	Derived from ID8-P	82
ID8-OR	Mouse	*Trp53*$^{-/-}$, *Brca2*$^{-/-}$	Olaparib-resistant. Derived from ID8-PB.	48
ID8-Scr	Mouse	*Trp53*$^{-/-}$, *Brca2*$^{-/-}$	Scrambled gRNA control. Derived from ID8-PB-OR	
ID8-*Rad52*KO#1, *Rad52*KO#2	Mouse	*Trp53*$^{-/-}$, *Brca2*$^{-/-}$, *Rad52*^{KO}	Rad52-knockout clone #1 and #2 via CRISPR/Cas9. Derived from ID8-PB-OR	
HGS2	Mouse	*Trp53*$^{-/-}$, *Brca2*$^{-/-}$, *Pten*$^{-/-}$		83
HGS2-OR	Mouse	*Trp53*$^{-/-}$, *Brca2*$^{-/-}$, *Pten*$^{-/-}$	Olaparib-resistant. Derived from HGS2	49
HGS2-Scr	Mouse	*Trp53*$^{-/-}$, *Brca2*$^{-/-}$, *Pten*$^{-/-}$	Non-targeting shRNA control. Derived from HGS2-OR	
HGS2-*Rad52*KD#1	Mouse	*Trp53*$^{-/-}$, *Brca2*$^{-/-}$, *Pten*$^{-/-}$	Rad52-knockdown clone #1. Derived from HGS2-OR	
